# The Obstetric Gazette

**Published:** 1878-08

**Authors:** 


The OBSTETRIC GAZETTE, Vol. 1.—No. 1, July, 1878 is the title of a new monthly, edited by Edward B. Stevens, M. D., of Lebanon, Ohio, and is devoted to Obstetrics and Diseases of women and children. Dr. Stevens has had considerable practice in the editorial chair, being connected for some years with the *Cincinnati Lancet*, and is therefore ably qualified for the work he has undertaken. The first number is rich in articles of practical value and we can heartily extend to the editor our congratulations and hope that it may prove a grand success.

